# Impact of Physical Interventions, Phosphorus Fertilization, and the Utilization of Soil Amendments on the Absorption of Cadmium by Lettuce Grown in a Solar-Powered Greenhouse

**DOI:** 10.3390/biology13050332

**Published:** 2024-05-10

**Authors:** Jun’an Zhang, Yingjun Hao, Guangsen Xiong, Quanzhong Tang, Xiwang Tang

**Affiliations:** 1 Hebei Engineering Research Center for Ecological Restoration of Rivers and Coastal Areas, Hebei University of Environmental Engineering, Qinhuangdao 066102, China; zhangjunan@hebuee.edu.cn (J.Z.); haoyingjun@hebuee.edu.cn (Y.H.); xiongguangsen@hebuee.edu.cn (G.X.); 2 Hebei Key Laboratory of Agroecological Safety, Hebei University of Environmental Engineering, Qinhuangdao 066102, China; 3 Department of Sociology, HSE University, Saint Petersburg 192148, Russia; qtang@hse.ru

**Keywords:** cadmium pollution, facility agriculture, lettuce, field trials, physical measures, phosphorus fertilizer, biochar, nano-hydroxyapatite, attapulgite clay

## Abstract

**Simple Summary:**

To find improved solutions for controlling cadmium pollution in agricultural soil, in situ field experiments were conducted to evaluate the impact of physical measures, phosphate fertilizer application, and soil conditioners on lettuce growth and cadmium absorption. The results revealed that deep plowing and soil covering significantly lowered the cadmium concentration in lettuce. While the application of phosphate fertilizer raised the cadmium concentration in lettuce, the use of diammonium phosphate and calcium magnesium phosphate fertilizer showed a positive correlation with the cadmium concentration, whereas calcium superphosphate displayed a negative correlation. Additionally, upon the application of biochar, attapulgite, and nano-hydroxyapatite, the cadmium concentration in lettuce initially increased with the rising amount of application, but subsequently decreased at higher doses. In conclusion, physical measures are evidently effective in preventing and controlling cadmium pollution. However, the application of phosphate fertilizer can exacerbate cadmium pollution in farmland soil. Soil conditioners, on the other hand, are only effective when used at higher application levels. The findings of this study are crucial for guiding practical cadmium pollution control measures in the field.

**Abstract:**

This study aimed to evaluate the effects of physical measures and the applications of phosphorus fertilizer and soil conditioner on the growth of lettuce (Lactuca sativa) and its uptake of cadmium (Cd). In a solar greenhouse that contained soil enriched with cadmium (Cd) (1.75 ± 0.41 mg/kg) with lettuce used as a test plant, field experimental methods were utilized to explore the influence of physical measures, such as deep plowing and soil covering, and the applications of phosphorus fertilizer, including diammonium phosphate (DAP), calcium magnesium phosphate (CMP), and calcium superphosphate (SSP), and soil conditioners, such as biochar, attapulgite clay, and nano-hydroxyapatite, on the uptake of Cd in lettuce. The results indicated that the concentrations of Cd in the aboveground parts of lettuce were 1.49 ± 0.45, 1.26 ± 0.02, 1.00 ± 0.21, and 0.24 ± 0.13 mg/kg when the soil was plowed 30, 40, and 50 cm deep, respectively, and when the soil was covered with 15 cm, this resulted in reductions of 27.5%, 38.3%, 51.4%, and 88.4%, respectively, compared with the control treatment that entailed plowing to 15 cm. When 75, 150, and 225 kg/ha of phosphorus pentoxide (P_2_O_5_) were applied compared with the lack of application, the contents of Cd in the aboveground parts of lettuce increased by 2.0%, 54.5%, and 73.7%, respectively, when DAP was applied; by 52.5%, 48.5%, and 8.1%, respectively, when CMP was applied; and by 13.1%, 61.6%, and 90.9%, respectively, when SSP was applied. When the amounts of biochar applied were 0, 2, 4, 6, 8, 10, and 12 t/ha, the contents of Cd in the aboveground parts of lettuce were 1.36 ± 0.27, 1.47 ± 0.56, 1.80 ± 0.73, 1.96 ± 0.12, 1.89 ± 0.52, 1.44 ± 0.30, and 1.10 ± 0.27 mg/kg, respectively. Under concentrations of 0, 40, 80, 120, 160, and 200 kg/ha, the application of nano-hydroxyapatite resulted in Cd contents of 1.34 ± 0.56, 1.47 ± 0.10, 1.60 ± 0.44, 1.70 ± 0.21, 1.31 ± 0.09, and 1.51 ± 0.34 mg/kg, respectively. The concentrations of Cd in the aboveground parts of lettuce treated with attapulgite clay were 1.44 ± 0.48, 1.88 ± 0.67, 2.10 ± 0.80, 2.24 ± 0.75, 1.78 ± 0.41, and 1.88 ± 0.48 mg/kg, respectively. In summary, under the conditions in this study, deep plowing and soil covering measures can reduce the concentration of Cd in the aboveground parts of lettuce. The application of phosphorus fertilizer increased the concentration of Cd in the aboveground parts of lettuce. The application of higher amounts of DAP and SSP led to greater concentrations of Cd in the aboveground parts of lettuce. The application of higher amounts of CMP caused a lower concentration of Cd in the aboveground parts of lettuce. When biochar, attapulgite clay, and nano-hydroxyapatite were applied, the concentration of Cd in the aboveground parts of lettuce increased in parallel with the increase in the concentration of application when low amounts were applied. In contrast, when high amounts were applied, the concentration of Cd in the aboveground parts of lettuce began to decrease.

## 1. Introduction

Facility agriculture is a high-input and high-output agricultural production model. Compared with ordinary open-field cultivation, it results in high yields and can produce vegetables out of season. Therefore, it has a higher output value and has been vigorously developed around the world, particularly in areas that are cold in the winter [1,2,3]. The higher rates of return have inspired farmers to apply large amounts of chemical fertilizers, organic fertilizers, and pesticides in facility agriculture production and adopt high-intensity planting methods, which result in a higher degree of intensive soil pollution than when the crops are cultivated in open fields [4,5,6,7,8,9,10,11]. Studies have shown that the content of elemental cadmium (Cd) in the soil of facility farmland in China currently exceeds the standard by much higher levels, and the degree of excess primarily exceeds the national standard in moderate and mild levels [9,12,13,14,15]. Cd is not essential for plants. Excessive amounts of Cd in the soil serve as an adverse factor for plant growth. When the concentration of Cd in the soil exceeds a certain limit, it can inhibit the growth of plants [16,17,18]. As a type of vegetable that is widely consumed around the world, lettuce (Lactuca sativa) can be enriched in Cd and have a high tolerance to Cd in the soil [15,18,19,20,21,22,23,24,25,26,27], which results in plants that can contain excessive amounts of hidden Cd. Cd easily accumulates in human tissues through the food chain, and this can endanger the health of the population. Thus, it is highly critical to conduct research on the prevention and control of the uptake of Cd in lettuce.

There are numerous measures to prevent and control the risk of soil pollution by Cd. Typical agricultural practices, such as plowing and covering the soil, can reduce the content of Cd contaminants on the soil surface, thereby mitigating the risk of Cd accumulation in vegetables [28]. Appropriate fertilization measures can decrease the risk of the pollution of soil with Cd by regulating its bioavailability in the soil [19,27,29,30,31]. The addition of conditioners to the soil can also mitigate the risk of soil becoming polluted with Cd. Moreover, the application of biochar, highly adsorbable clay, and nanomaterials can also prevent and control Cd pollution [20,23,25,32,33]. However, most studies on the prevention and control of Cd pollution have been conducted in pots, and there have only been limited results from field trials [34]. The prevention and control of Cd pollution measures merit the evaluation of more field trials. This study aimed to evaluate the effects of physical measures and the applications of phosphorus fertilizer and soil conditioners on the growth of lettuce and its uptake of Cd. To reduce the risk of Cd pollution in a solar greenhouse where Cd is enriched owing to long-term high-intensity cultivation, field experiments were conducted to explore the prevention and control effects of deep plowing and soil covering and the application of phosphorus fertilizer, biochar, attapulgite clay, and nano-hydroxyapatite on the risk of soil Cd pollution in solar greenhouses. These results can provide a scientific basis to control the risk of Cd pollution during the production process of lettuce in facility agriculture and are highly significant for ensuring the safe production of lettuce in facility agriculture.

## 2. Materials and Methods

### 2.1. Overview of the Experimental Site

The experimental site was located in Nanying Village, Wuquan Town, Yangling District, Shaanxi Province, China, with an annual precipitation of approximately 600 mm, an average annual temperature of 13.6 °C, and an average amount of annual radiation of 440–544 kJ/cm^2^. The solar greenhouse used in the experiment was a northwest type. The soil was Lou type composed of heavy loam with a pH of 7.83 ± 0.10. This was true for both the 0–15 cm cultivated layer and the soil below. The soil contained 36.12 ± 1.2 g/kg of organic matter, total nitrogen of 1.72 ± 0.06 g/kg, available phosphorus of 302.6 ± 8.52 mg/kg, available potassium of 721.6 ± 14.39 mg/kg, a CEC of 24.21 ± 2 cmol^+^ /kg, and 1.75 ± 0.41 mg/kg of Cd.

### 2.2. Experimental Design

The physical measures included deep plowing and soil covering. Gradients were utilized for the depth of plowing, including 15, 30, 40, and 50 cm. The plowing depth was uniformly plowed by human labor. The customary plowing depth (CK) was 15 cm. Only one treatment level was established for the soil covering treatment that was 15 cm thick. The soil for the covering treatment was obtained from a greenhouse in a nearby facility that was not contaminated by Cd (0.24 ± 0.15 mg/kg).

The phosphorus fertilizers tested were diammonium phosphate (DAP), calcium magnesium phosphate (CMP), and calcium superphosphate (SSP). They were purchased from local agricultural material supply and marketing cooperatives. The contents of phosphorus in terms of P_2_O_5_ were 44%, 12.5%, and 46%, respectively. Four concentrations of phosphorus fertilizer levels were established, including 0, 75, 150, and 225 kg/ha. The concentration of Cd in the phosphorus fertilizers was less than 10 mg/kg.

The soil conditioners that were tested were biochar, attapulgite clay, and nano-hydroxyapatite. The biochar (heated temperature was 300 °C) was prepared from corn straw and purchased from the Beijing Academy of Agricultural Sciences (Beijing, China). The pH value was 8.45. Six levels of application were established, including 0, 2, 4, 6, 8, 10, and 12 t/ha. The particle size of attapulgite clay was 7 μm, and it was produced in Mingguang County, Anhui Province, China. The nano-hydroxyapatite was analytical grade and purchased from Shanghai Yuanye Biotechnology Co., Ltd. (Shanghai, China). The concentration gradients of hydroxyapatite and attapulgite clay that were applied were 0, 40, 80, 120, 160, and 200 kg/ha.

### 2.3. Field Experiments

The field plots were arranged in random blocks, with blocks that were parallel to the long side of the solar greenhouse. Three replicates were established for each treatment. The area of each plot was 2 × 1.5 m^2^, with a plot spacing of 20 cm and a block spacing of 30 cm. A mini-tiller was used to thoroughly mix the soil in the tillage layer of the experimental plots before they were divided. In the plots treated with deep plowing and soil covering measures, the lettuce was cultivated after various treatments. In the plots treated with phosphorus fertilizers and soil conditioners, the phosphorus fertilizers and soil conditioners were spread evenly over the soil surface of the plot to mix evenly with the soil in the tillage layer before the lettuce was planted. Subsequently, the lettuce was planted and cultivated. The variety utilized was Lisheng lettuce with the trade name “Lisheng No. 2.” The biomass per plant was 300–500 g, and the plants were grown for 70 d [15]. The lettuce was transplanted and planted after seedlings had been grown in the field. The seedlings were transplanted when the Lisheng No. 2 lettuce grew its fourth leaf. A total of 30 plants were planted evenly in each plot. Samples of the lettuce were collected and measured at 60 d after planting.

### 2.4. Analytical Methods

#### 2.4.1. Determination of the Chlorophyll and Plant Height

Five lettuce samples were randomly selected from each plot. The content of the chlorophyll in the mature leaves was measured using a SPAD-502 chlorophyll rapid tester (Spectrum Technologies, Aurora, IL, USA). During the measurements, multiple points of the same lettuce were measured, and the average value was recorded. Moreover, the height of the selected lettuce plants was measured using a ruler. Each lettuce plant was measured using the same method.

#### 2.4.2. Determination of the Aboveground Biomass

Stainless steel knives were used to collect the aboveground parts of the lettuce plants selected. These parts were washed sequentially with tap water, deionized water, and high-purity water, dried, and then weighed to determine their fresh weight. They were subsequently cured in an oven at 105 °C for 1 h and dried at 75 °C to constant weight, followed by dry weight measurement. The dried samples were crushed and ground into fine pieces and placed in polyethylene Ziplock bags for storage until testing.

#### 2.4.3. Determination of the Levels of Cd

The lettuce samples stored in polyethylene Ziplock bags were digested using the nitric acid–perchloric acid (HNO_3_-HClO_4_) (3:1) method at 160 °C with an electric hot plate. The Cd in the samples was measured using a Hitachi Z-2000 atomic absorption spectrometer (AAS) (Hitachi, Tokyo, Japan) (Tang et al., 2016, [15]). During the process of sample analysis, environmental standard material GBW-10015 (spinach [Spinacea oleracea]) was used for quality control to ensure a recovery rate of 95 ± 5%, with two replicates per batch.

#### 2.4.4. Data Analysis

Data were analyzed using Microsoft Excel 2010 (Microsoft Corp., Redmond, WA, USA) and SigmaPlot 12.5 (Systat Software Inc., San Jose, CA, USA). One-way ANOVA followed by Duncan’s multiple range test was conducted at α = 0.05. *p* < 0.05 was considered significant. Figures were made using Python 3.10.

## 3. Results

### 3.1. Effects of the Various Treatments on Lettuce Growth

The fresh weight, chlorophyll, plant height, and leaf width of the aboveground single plant of lettuce were measured as indicators to evaluate the growth status. The results are shown in Table 1, Table 2 and Table 3. The response of growth to each treatment was obtained by analyzing the changes in various growth indicators of lettuce under each treatment.

#### 3.1.1. Deep Plowing and Soil Covering

Table 1 revealed that the fresh weight of a single aboveground lettuce plant in the deep plowing and soil covering treatments ranged from 170 to 191 g; the plant height ranged from 27.5 to 27.7 cm; the leaf width was between 67.4 and 74.9 mm; and the chlorophyll was between 28.4 and 29.6 SPAD^−1^. Except for the chlorophyll, all the indicators of lettuce treated with deep plowing and soil covering were significantly lower than those of the CK treatment (*p* < 0.05).

**Table 1:** Lettuce biomass, chlorophyll, plant height, and leaf length under soil physical measures applications to remediate cadmium (Duncan’s Method, *n* = 15, α = 0.05).

Soil Cd Remediation Applications			Lettuce Biomass (g/plant)	Chlorophyll (SPAD)	Plant Height (cm)	Leaf Length (mm)
Deep plowing and soil covering (cm)	Deep plowing	15 (CK)	279 ± 43 a	29.6 ± 2.7 a	29.4 ± 0.7 a	84.3 ± 3.9 a
		30	191 ± 13 b	28.7 ± 3.5 a	27.7 ± 0.4 b	73.7 ± 4.3 b
		40	166 ± 31 b	28.4 ± 3.2 a	27.5 ± 0.3 b	67.4 ± 4.7 b
		50	151 ± 24 b	26.9 ± 2.2 a	27.7 ± 0.5 b	74.9 ± 3.3 b
	Soil covering	15	170 ± 33 b	28.5 ± 2.4 a	27.6 ± 0.6 b	70.5 ± 2.7 b

Note: the small letters appearing after the value of the standard deviation stands for the result of Duncan’s multiple range test. Data with same letter means they were no significant difference at the value α = 0.05.

#### 3.1.2. Application of Phosphorus Fertilizers

Table 2 shows that the fresh weight of lettuce plants treated with the three phosphorus fertilizers ranged from 198 to 321 g/plant. When DAP and SSP were applied to the soil, the fresh weight of a single aboveground lettuce plant increased with an increase in the concentration of phosphorus fertilizers. When CMP phosphate fertilizer was applied to the soil, the fresh weight of a single aboveground lettuce plant decreased with an increase in the concentration of phosphorus fertilizer applied. Among them, the fresh weight of the aboveground single plant of lettuce treated with P_2_O_5_ of 225 kg/ha CMP fertilizer was significantly lower than that of the other treatments. The width of lettuce leaves in each treatment fluctuated between 75 and 84 mm, and the width of leaves treated with CMP fertilizer was significantly lower than that of other treatments. No significant differences in chlorophyll and plant height were observed between the treatments.

**Table 2:** Lettuce biomass, chlorophyll, plant height, and leaf length under soil phosphorus fertilizer applications to remediate cadmium (Duncan’s Method, *n* = 15, α = 0.05).

Soil Cd Remediation Applications			Lettuce Biomass (g/plant)	Chlorophyll (SPAD)	Plant Height (cm)	Leaf Length (mm)
Phosphorus fertilizer application rates (P_2_O_5_ kg/ha)	DAP	75	272 ± 26 a	33 ± 0.6 a	30.7 ± 0.9 a	83 ± 3.8 a
		150	286 ± 18 a	32 ± 2.4 a	32.4 ± 3.3 a	82 ± 2.0 a
		225	310 ± 22 a	34 ± 2.2 a	30.4 ± 2.1 a	84 ± 2.2 a
	CMP	75	272 ± 16 a	30 ± 1.9 a	28.5 ± 1.2 a	75 ± 3.0 b
		150	264 ± 23 a	31 ± 3.2 a	28.7 ± 1.5 a	75 ± 3.0 b
		225	198 ± 18 b	30 ± 0.1 a	29.5 ± 0.6 a	76 ± 2.4 b
	SSP	75	265 ± 21 a	31 ± 2.9 a	30.1 ± 0.8 a	81 ± 2.8 a
		150	296 ± 23 a	30 ± 2.1 a	30.9 ± 0.9 a	84 ± 3.1 a
		225	312 ± 34 a	29 ± 2.6 a	29.9 ± 0.5 a	82 ± 3.0 a
	CK	0	279 ± 33 a	30 ± 1.3 a	29.4 ± 0.2 a	84 ± 3.6 a

Note: CK, control; CMP, calcium magnesium phosphate; DAP, diammonium phosphate; P_2_O_5_, phosphorus pentoxide; SSP, calcium superphosphate. The small letters appearing after the value of the standard deviation stands for the result of Duncan’s multiple range test. Data with same letter means they were no significant difference at the value α = 0.05.

#### 3.1.3. Application of the Soil Conditioners

Table 3 shows the lettuce growth status with the application of the soil conditioners. When biochar was applied to the soil, the aboveground biomass of a single lettuce plant ranged from 162 to 181 g. In the control treatment, the aboveground biomass of lettuce was 149 g/plant. The results indicated that biomass in the biochar treatment was significantly higher than that of the control treatment. Compared with a lack of biochar, its application increased the biomass of individual plants in the aboveground parts of lettuce but had no significant effect on its plant height, chlorophyll, and leaf width.

There were no significant differences in the aboveground fresh weight, plant height, chlorophyll, and leaf width of lettuce between the treatments of attapulgite clay and nano-hydroxyapatite applied to the soil and compared with the control treatment. The effects of attapulgite clay and nano-hydroxyapatite on lettuce growth were statistically insignificant (*p* > 0.05).

**Table 3:** Lettuce biomass, chlorophyll, plant height, and leaf length under soil conditioner applications to remediate cadmium (Duncan’s Method, *n* = 15, α = 0.05).

Soil Cd Remediation Applications			Lettuce Biomass (g/plant)	Chlorophyll (SPAD)	Plant Height (cm)	Leaf Length (mm)
Soil amendment	Biochar (t/ha)	2	177 ± 23 ab	28.6 ± 2.4 a	29.2 ± 2.1 a	70.6 ± 4.6 a
		4	171 ± 24 ab	29.1 ± 3.0 a	28.4 ± 3.5 a	67.5 ± 6.4 a
		6	160 ± 33 ab	29.4 ± 4.3 a	26.7 ± 2.0 a	65.2 ± 6.1 a
		8	162 ± 29 ab	28.5 ± 3.1 a	27.3 ± 1.9 a	69.9 ± 8.0 a
		10	173 ± 20 ab	27.3 ± 4.3 a	27.8 ± 1.9 a	68.0 ± 14.0 a
		12	181 ± 17 a	27.7 ± 3.4 a	27.4 ± 1.9 a	65.6 ± 8.5 a
		CK	149 ± 22 b	28.1 ± 1.8 a	26.5 ± 2.3 a	65.2 ± 11.1 a
	Attapulgite clay (kg/ha)	0 (CK)	229 ± 38 a	32.7 ± 2.6 a	25.7 ± 2.3 a	71.4 ± 10 a
		40	249 ± 67 a	31.5 ± 3.9 a	26.0 ± 2.5 a	75.5 ± 7.2 a
		80	267 ± 105 a	31.8 ± 3.0 a	26.9 ± 4.0 a	74.2 ± 9.9 a
		120	227 ± 28 a	31.6 ± 2.3 a	26.2 ± 2.5 a	71.0 ± 8.7 a
		160	245 ± 19 a	32.2 ± 3.6 a	28.2 ± 2.4 a	74.2 ± 7.6 a
		200	233 ± 70 a	32.0 ± 2.3 a	26.3 ± 3.2 a	71.1 ± 7.9 a
	Nano-hydroxyapatite (kg/ha)	0 (CK)	248 ± 48 a	32.5 ± 3.0 a	27.4 ± 3.6 a	69.1 ± 8.2 a
		40	213 ± 67 a	33.1 ± 3.9 a	25.9 ± 4.3 a	67.3 ± 9.3 a
		80	225 ± 73 a	32.8 ± 3.1 a	25.7 ± 3.4 a	68.7 ± 11.1 a
		120	235 ± 57 a	32.3 ± 3.0 a	26.2 ± 3.6 a	73.4 ± 8.5 a
		160	214 ± 68 a	32.4 ± 3.2 a	26.7 ± 2.6 a	69.0 ± 11.1 a
		200	202 ± 60 a	32.4 ± 3.1 a	24.9 ± 3.9 a	65.7 ± 9.8 a

Note: The small letters appearing after the value of the standard deviation stands for the result of Duncan’s multiple range test. Data with same letter means they were no significant difference at the value α = 0.05.

### 3.2. Effects of Various Treatments on the Uptake of Cd in Lettuce

#### 3.2.1. Physical Measures

**Figure 1:**
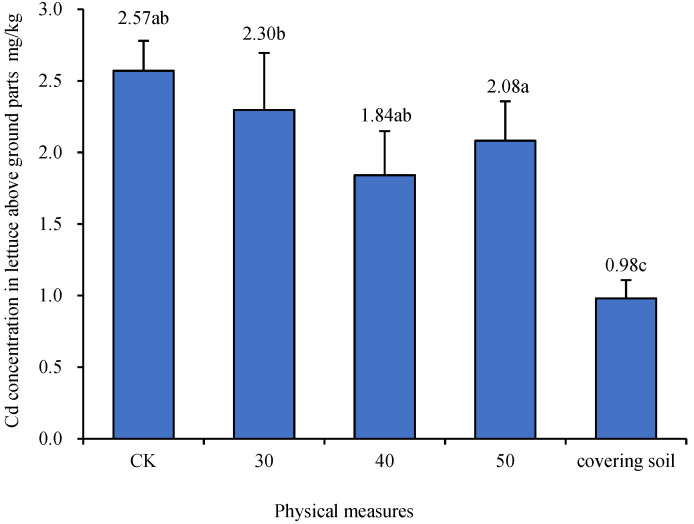
The concentration of cadmium (Cd) in the aboveground parts of lettuce after physical measures. CK, 30, 40, 50, and covering soil represent plowing treatments of 15, 30, 40, and 50 cm and 15 cm of surface covering soil, respectively (Duncan’s Method, *n* = 3, α = 0.05). The small letters appearing in figure caption stands for the result of Duncan’s multiple range test. Data with same letter means they were no significant difference at the value α = 0.05.

The results of physical measures are shown in Figure 1. The concentrations of Cd in the aboveground parts of lettuce after deep plowing of 30, 40, and 50 cm were 2.30, 1.84, and 2.08 mg/kg, respectively. The concentration of Cd in the aboveground parts of lettuce in the soil covering treatment was 0.98 mg/kg. Compared with the CK treatment, the concentrations of Cd were reduced by 10.7%, 28.4%, 19.0%, and 61.8%, respectively. In the 40 cm plowing treatment, the concentration of Cd in the aboveground parts of lettuce was significantly lower (*p* < 0.05) than that in the CK treatment and significantly higher (*p* < 0.05) than that in the soil covering treatment. The concentration of Cd in the aboveground parts of lettuce treated with deep plowing of 30 cm and deep plowing of 50 cm was lower than that of the CK treatment and higher than that of the deep plowing treatment of 40 cm, whereas the differences did not reach a significant level (*p* > 0.05).

#### 3.2.2. Applications of Phosphorous

**Figure 2:**
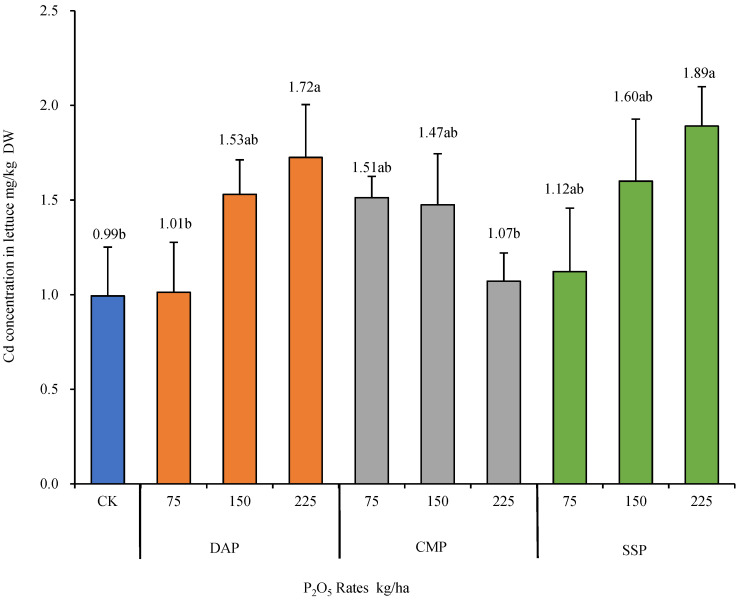
The concentration of cadmium in the aboveground parts of lettuce under different rates of applications of P_2_O_5_ (Duncan’s Method, *n* = 3, α = 0.05). CMP, calcium magnesium phosphate; DAP, diammonium phosphate; P_2_O_5_, phosphorus pentoxide; SSP, calcium superphosphate. The small letters appearing in figure caption stands for the result of Duncan’s multiple range test. Data with same letter means they were no significant difference at the value α = 0.05.

The concentration of Cd in the aboveground parts of lettuce under different phosphorus fertilizer treatments is shown in Figure 2. Compared with no fertilization, the application of three types of phosphorus fertilizers increased the concentration of Cd in the aboveground parts of lettuce to varying degrees. The application of DAP promoted the uptake of Cd in the aboveground parts of lettuce. When the rates of application were 75, 150, and 225 kg/ha P_2_O_5_, the contents of Cd in the aboveground parts of lettuce were 1.01, 1.53, and 1.72 mg/kg, respectively. Compared with the concentration of 75 kg/ha P_2_O_5_, those of 150 and 225 kg/ha P_2_O_5_ increased the concentration of Cd in the aboveground parts of lettuce by 51.5% and 70.3%, respectively. The application of CMP fertilizers reduced the uptake of Cd in the aboveground parts of lettuce. When the rates of application were 75, 150, and 225 kg/ha P_2_O_5_, the contents of Cd in the aboveground parts of lettuce were 1.51, 1.47, and 1.07 mg/kg, respectively. When SSP was applied at 75, 150, and 225 kg/ha P_2_O_5_, the contents of Cd in the aboveground parts of lettuce were 1.12, 1.60, and 1.89 mg/kg, respectively. As the dosage of SSP increased, the concentration of Cd in the aboveground parts of lettuce also increased.

#### 3.2.3. Application of Soil Conditioners

**Figure 3:**
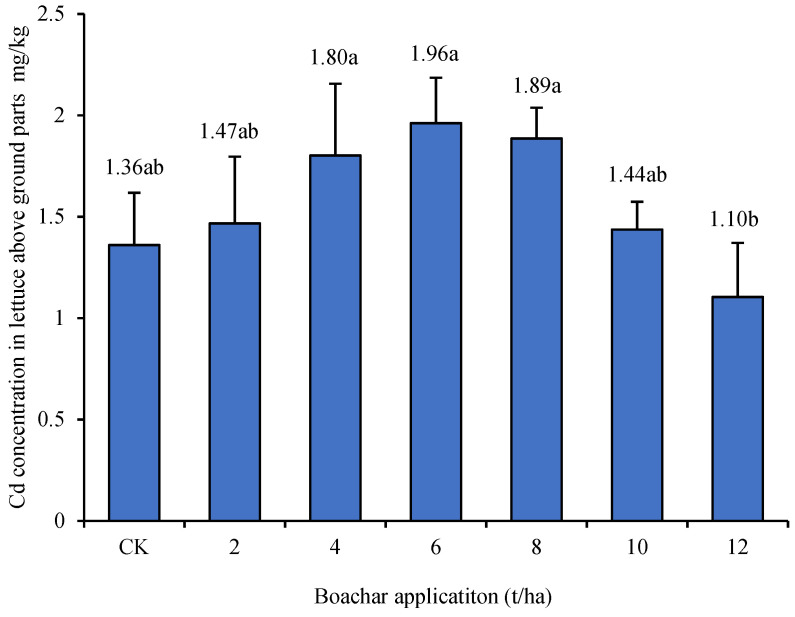
Effect of biochar on the uptake of cadmium (Cd) in the aboveground parts of lettuce (Duncan’s Method, *n* = 3, α = 0.05). The small letters appearing in figure caption stands for the result of Duncan’s multiple range test. Data with same letter means they were no significant difference at the value α = 0.05.

The content of Cd in the aboveground parts of lettuce with biochar applied to the soil is shown in Figure 3. As the amounts of biochar increased, the content of Cd in the aboveground parts of lettuce first increased and then decreased. When 6 t/ha of biochar was applied, the content of Cd in the aboveground parts of lettuce reached a maximum value of 1.96 mg/kg. When < 6 t/ha of biochar was applied, compared with the control treatment, the content of Cd increased by 8.1%, 32.4%, and 44.1%, respectively, under the biochar additions of 2, 4, and 6 t/ha. When > 6 t/ha was applied, compared with the rate of biochar application of 6 t/ha, the contents of Cd decreased by 3.6%, 26.5%, and 43.0%, respectively, under the concentrations of biochar of 8, 10, and 12 t/ha. Among them, when biochar was applied at 12 t/ha, the content of Cd in the aboveground parts of lettuce was significantly lower than that of the 6 t/ha treatment.

**Figure 4:**
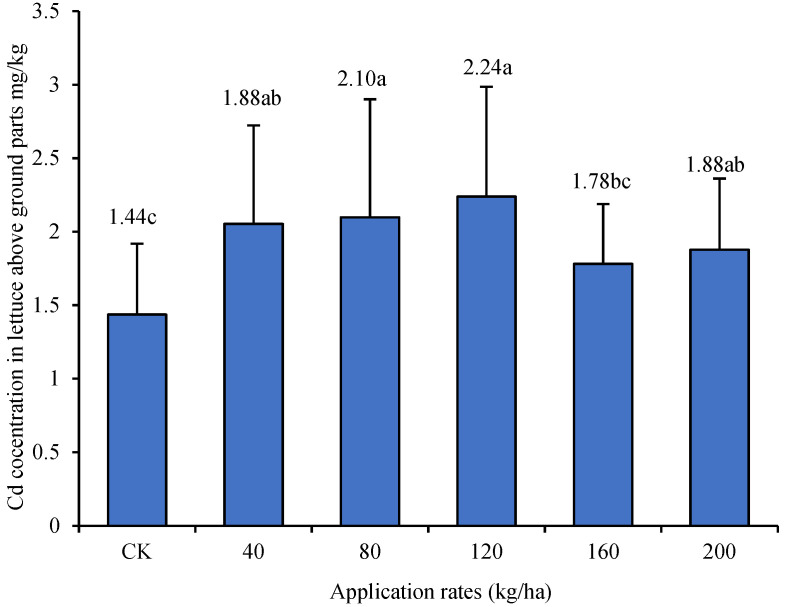
Effect of the application of attapulgite clay on the uptake of cadmium (Cd) by lettuce (Duncan’s Method, *n* = 3, α = 0.05). The small letters appearing in figure caption stands for the result of Duncan’s multiple range test. Data with same letter means they were no significant difference at the value α = 0.05.

The results of the application of attapulgite clay are shown in Figure 4. The contents of Cd in the aboveground parts of lettuce treated with 0, 40, 80, 120, 160, and 200 kg/ha of attapulgite clay were 1.44, 1.88, 2.10, 2.24, 1.78, and 1.88 mg/kg, respectively. The contents of Cd in the aboveground parts of lettuce treated with attapulgite clay were higher than those without application. The differences were significant (*p* < 0.05) except for the rate of application of 160 kg/ha.

**Figure 5:**
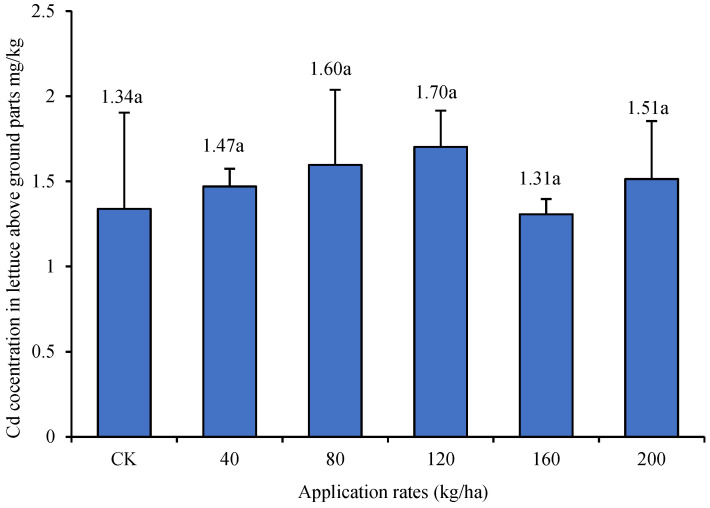
Effect of nano-hydroxyapatite applications on the uptake of cadmium (Cd) in lettuce (Duncan’s Method, *n* = 3, α = 0.05). The small letters appearing in figure caption stands for the result of Duncan’s multiple range test. Data with same letter means they were no significant difference at the value α = 0.05.

The results of the application of nano-hydroxyapatite are shown in Figure 5. The contents of Cd in the aboveground parts of lettuce after the application of 0 (CK), 40, 80, 120, 160, and 200 kg/ha of nano-hydroxyapatite were 1.34, 1.47, 1.60, 1.70, 1.31, and 1.51 mg/kg, respectively. There was no significant difference in the aboveground content of Cd in lettuce between treatments with different concentrations of nano-hydroxyapatite and the CK. As the concentration of nano-hydroxyapatite increased from 0 kg/ha to 120 kg/ha, the content of Cd in the aboveground parts of lettuce tended to increase. When the content of nano-hydroxyapatite > 120 kg/ha, the content of Cd in the aboveground parts of lettuce began to decrease.

## 4. Discussion

Both deep plowing and soil covering measures significantly reduced the concentration of Cd in the aboveground parts of lettuce. This is because the Cd in solar greenhouse soil primarily accumulates on the soil surface [4,6,7,11]. After deep plowing, the concentration of Cd in the surface layer of the mixed soil was reduced, thereby mitigating its harm. However, deep plowing and soil covering measures weakened the growth of lettuce, and the impact on lettuce biomass was more significant. Generally, the long-term shallow plowing of soil after deep plowing facilitates the permeability of the soil to water and air, which is conducive to an increase in crop yields [35,36]. The reason for the different results observed in this study is that the soil in the solar greenhouse had been shallowly tilled for a long time and had formed a plow bottom layer. Consequently, the soil fertility below the tillage layer was poor, and the improvement of soil tiltability could not make up for the impact of the lack of fertility on lettuce growth. Thus, when using deep plowing and soil covering measures to prevent and control the soil pollution of Cd in facilities, attention should be paid to assessing the impact on soil fertility.

Phosphorus fertilizer is one of the three primary types of fertilizers. In numerous studies, phosphate, as a solidifying agent for soil Cd, can increase the content of residual Cd in the soil, thereby effectively reducing its bioavailability in the soil and alleviating its risk of pollution [31,37,38]. However, this study observed that the application of phosphorus fertilizer increased the concentration of Cd in the aboveground parts of lettuce compared with the lack of application of fertilizer. This study also observed that as the concentrations of phosphorus fertilizer increased in the field experiment, the changes in the fresh weight of the aboveground single lettuce plants maintained a consistent trend with the changes in Cd concentration, indicating that phosphorus fertilizer may influence the uptake of Cd by lettuce by affecting the accumulation of lettuce biomass. Moreover, this study observed that the application of CMP fertilizers increased the concentration of Cd in the aboveground parts of lettuce. However, as the concentrations of CMP fertilizers increased, the concentration of Cd in the aboveground parts of lettuce decreased, and the growth indicators of lettuce also decreased. This is similar to the results of some field experiments. After the application of phosphorus fertilizers, phosphate fertilizers increased the biomass of the test plants, thereby enhancing the plant’s uptake of Cd [39,40,41,42]. Alternatively, the soil pH of the experimental plot in this study was 7.83, and the soil was calcareous. The application of phosphate may not have a strong effect on the solidification of Cd in the soil. In contrast, owing to the low availability of phosphorus in alkaline soil, the application of phosphorus fertilizer may promote the growth of lettuce, consequently improving the uptake of Cd.

Similarly, the effects of the application of biochar, nano-hydroxyapatite, and attapulgite clay on the prevention and control of Cd were greatly reduced owing to the excessively high pH of the field soil in this experiment. Biochar can improve the properties of soil, enhance the soil rhizosphere microbial environments, and also reduce the uptake of Cd by test plants at higher rates of application [20,25,26,33]. In this study, the ability of biochar to improve the soil was more apparent. The aboveground biomass of lettuce was significantly higher than that in the absence of the application of biochar. At a low concentration, there was an increase in the content of Cd in the aboveground parts of lettuce. Nano-hydroxyapatite and attapulgite clay are both soil amendments. They possess enormous specific surface areas and adsorption properties. They can absorb soil heavy metals and improve soil. The two have similar effects after application to the soil [43,44]. However, the soil in the test field in this study was a calcareous alkaline soil, which has a high cation exchange capacity. The effect of nano-hydroxyapatite and attapulgite clay in preventing and controlling the risk of cadmium pollution was not apparent, but the effect of improving soil and promoting plant growth was achieved. In this study, it was also observed that at lower dosages, both nano-hydroxyapatite and attapulgite clay promoted the uptake of Cd in lettuce.

It should be noted that although this study obtained some useful results, there are still some limitations, such as the facts that the field trial was only conducted for one cycle, only one cadmium-contaminated field was tested, and only one type of lettuce was used as the test crop in the experiment, which may limit the applicability of the study results. In addition, researchers need to understand that field trials can be influenced by many factors, and some factors that we have not taken into account may affect the accuracy of the study results.

## 5. Conclusions

This study drew the following conclusions. (1) Physical resistance and control measures (deep plowing and soil covering) effectively reduced the concentration of Cd in the cultivated layer of facility agriculture soil and the uptake of Cd in the aboveground parts of lettuce. However, the impact on soil fertility merits consideration. (2) Under the conditions of this experimental field, the application of phosphorus fertilizer increased the uptake of Cd by lettuce compared with a lack of fertilizer. However, the amounts of DAP and SSP that were applied positively correlated with the concentration of Cd in the aboveground parts of lettuce, whereas the application of CMP fertilizer negatively correlated with the content of Cd in the aboveground parts of lettuce. (3) When biochar was applied, the concentration of Cd in the aboveground parts of lettuce had a trend of first increasing and then decreasing as the amounts that were applied increased. When low amounts of biochar were applied (<6 t/ha), the concentration of Cd in the aboveground parts of lettuce increased with the increase in the amounts of biochar applied. In contrast, the results were opposite when high amounts were applied (>6 t/ha). (4) When <120 kg/ha of nano-hydroxyapatite and attapulgite clay was applied, the concentration of Cd in the aboveground parts of lettuce increased with the amounts applied. The concentration of Cd in the aboveground parts of lettuce began to decrease at >120 kg/ha.

## Data Availability

All data generated or analyzed during this study are included in this published article.
